# The Causes, Nature, Diagnosis, and Treatment of Acute Hydrocephalus

**Published:** 1843-04

**Authors:** 


Art. VI.—*The Causes, Nature, Diagnosis, and Treatment of Acute Hydrocephalus.* By J. R. Bennett, m.d.—*London*, 1843. 8vo, pp. 254.

This work is the essay to which the Fothergillian gold medal was awarded by the London Medical Society; and it is well deserving a yet higher meed: it is a most excellent prize essay. The author displays an extensive acquaintance with the writings of his predecessors, whose opinions are always stated clearly and with candour, while his own are expressed with modesty. As a good digest of what is already known on the subject of hydrocephalus we can conscientiously recommend the essay to our readers, and would especially call their attention to the chapter on the statistics of the disease, which contains much valuable information on points which we do not recollect to have seen noticed in any English work on the subject. The statistics occupy the second chapter of the work; the first is devoted to a detail of the symptoms of hydrocephalus; the third, to a description of the post-mortem appearances; and the causes, nature, diagnosis, and treatment are discussed in successive chapters. All are evidently the production of a well-informed writer, and able physician; but in all we miss that impress of vitality which personal observation only can impart to a description of natural phenomena. There is, too, a constant use of the vague expressions *some*, *many*, *often*, which, when subjects are treated of that admit of being exhibited by numbers, is at the present day scarcely excusable. For this defect we were at a loss to account, until we found, at p. 92, that the number of post-mortem examinations made by Dr. Bennett was only ten; and from p. 62, it further appears probable that they are all the cases of which he has preserved notes. Now this may be all very well for a paper submitted to the council of the Medical Society, but when it meets us in a printed book we must criticise it. Our knowledge of a disease, confessedly so obscure, to the investigation of which men of great talent have devoted much time and labour, and have yet left many points in its pathology and treatment undetermined, is not likely to be promoted by a compilation, however well executed, of what is already familiar to the profession.

It is with no unfriendly spirit to Dr. Bennett that we make these observations, but because we are sure that if he will devote himself to the search "for truth in Truth's own book," and not content himself with being the copyist of others, he possesses every requisite for becoming a most successful cultivator of our art. We hope before long again to meet with him in the character of an independent observer. For the present, we wish him good speed, commending to his notice an apophthegm of one whose memory, we doubt not, he joins with us in revering, "Was sich im Umgang der Natur und in ihrem Anschauen entwickelt, hat mehr Werth als alles Erdachte oder Erlernte. Das allein hat nur wahres Leben, d. h. den Geist der Natur, und ist so ewig wahr wie sie."

